# Lumpy Skin Disease Virus *(LSDV)* recently emerged in Egypt: molecular detection, and assessment of the related hematobiochemical, and risk factors

**DOI:** 10.1186/s12917-026-05442-7

**Published:** 2026-04-13

**Authors:** Emad Beshir Ata, Ashraf Khamees Shaban, Fatma F. Mohamed, Marwa F. Mahmoud

**Affiliations:** 1 Parasitology and Animal Diseases Department, Veterinary Research Institute, National Research Centre (NRC), Dokki, Giza Egypt; 2 Virology Department, Animal Health Research Institute (AHRI) Shebeen El-Kome Branch, Agricultural Research Center (ARC), Shebeen El-Kome, Menoufia Egypt; 3 Biochemistry Department, Animal Health Research Institute (AHRI), Shebeen El- Kome Branch, Agricultural Research Center (ARC), Shebeen El-Kome, Menoufia Egypt; 4 Virology Unit, Animal Reproduction Research Institute (ARRI), Agriculture Research Center (ARC), Giza, Egypt

**Keywords:** LSDV, Molecular identification, DIVA qPCR, Phylogeny, Risk factors, Hematological parameters

## Abstract

The lumpy skin disease (LSD) is a notifiable disease caused by a DNA virus of the genus *Capripox.* Despite application of massive vaccination strategy, LSD still endemic in Egypt with emerging outbreaks and devastating outcome. Therefore, the target of this study was isolation and updated molecular characterization of the emerged circulating strains adding to evaluation of the related biochemical parameters, and risk factors. Tissue samples (168) were collected from infected cattle and used for virus isolation on MDBK, IFAT, molecular detection by PCR amplification, sequencing and phylogenetic analysis of both GPCR, and EEV glycoprotein genes. DIVA qPCR was applied to differentiate infected and vaccinated animals. Whole blood, and serum samples were obtained from infected and healthy animals to determine the correlated hematobiochemical parameters. The virus was successfully isolated from 38.69% (65/168) of the tissue samples and confirmed by IFAT. Molecular detection using the GPCR primer set was more sensitive either in the blood (67.26%) (113/168) or tissue samples (88.09%) (148/168). All of the detected virus strains were related to field isolates not vaccinal ones. The constructed phylogenetic tree showed the clear distinguishing of *capripox* lineages, relatedness of the obtained isolates to each other, and those circulating in Nigeria, and Vietnam. Sex, age, breed, and vaccination were the prominent significant factors. Hematologically, anemia, hypoproteinemia, significant increase in blood urea nitrogen, creatinine, AST, and ALT were recorded in relation with hematological, biochemical, oxidant, and antioxidant parameters changes. In conclusion, this study succeeded in isolation, and molecular detection of circulating *LSDV*. Also, it highlighted the related risk factors, and the importance of immunization, deep investigation to discover the mode of transmission rather than the known traditional ones.

**Supplementary Information:**

The online version contains supplementary material available at 10.1186/s12917-026-05442-7.

## Introduction

Animals play an important role in keeping the global food security [1–4]. They were affected by a variable range of pathogens that affect their productivity [5–8].

Lumpy skin disease (LSD) is a transboundary, emerging, and notifiable one affecting cattle all over the world. The causative virus (*LSDV*) is a double stranded DNA, a member of the *Capripoxvirus lumpyskinpox* [9]. The causative virus showed a high resistance to environmental conditions. Although, it is host-specific for cattle and some domestic water buffalo breeds. But wild ruminants could be potential reservoir for spreading the *LSDV* [10].

This disease poses great significance on animal productivity either directly through its high morbidity or mortality rates, and indirectly due to the occasional occurrence of mastitis, infertility, mortality, and decrease of milk production [11]. Previous results cleared association with multiple reproductive problems including abortion, temporary/permanent infertility (especially in bulls due to testicular damage), prolonged anestrus, poor body condition, during long-lasting fever that causes the absence of the estrous cycle [12, 13].

The clinical course of the disease ranges from subclinical through mild to acute stages, the affected animals develop different signs including fever, generalized skin nodules, and swollen lymph nodes [14], . But a recent study cleared that some infected cattle displayed no clinical signs [15].

Hematologically, the disease is characterised by inflammatory leukogram, anemia, and altered kidney function [16]. In late stage, hemolytic anemia, leukocytosis, disturbance in liver and kidney function were recorded [17].

Although LSD has characteristic clinical lesions, accurate diagnosis is mandatory for effective control. Isolation of virus is the standard method, but it is labor and time consuming. Therefore, the different molecular techniques were extensively used not for accurate identification but also for its high sensitivity, and the subsequent phylogenetic analysis to trace back its origin and relatedness to other *Capri pox viruses* [18]. The virus was successfully detected in different samples like blood, tissue, and semen specimens using specific primers for different genes but those of GPCR, and EEV glycoprotein genes were extensively used [19, 20].

Inappropriate usage of vaccines and the partial protection of certain ones compromise the effectiveness of vaccination-based LSD control strategy [21]. Understanding the genetic diversity of field isolates during outbreaks necessitates the genetic characterization of *LSDV* [22]. However, misused vaccines can cause vaccine strains to recombine and co-infect with virulent strains, causing virulent reversal of vaccine strains and potentially causing additional outbreaks [23]. Therefore, the goal of this research was not only characterization of the emergent *LSDV* in infected cattle during outbreaks in Egyptian localities from 2023 to 2025 through virological and molecular techniques including conventional PCR, sequencing, and phylogeny, but also DIVA qPCR was used for determination of the causative agent either due to field strains or evolved from the used vaccines. Furthermore, some Immuno-biochemical parameters, and related risk factors were studied.

## Materials and methods

### Ethical approval

All the animal experiments were carried out according to the Animal Welfare directives. The present study was ethically approved by the Ethics Committee of the Animal Reproduction Research Institute (Approval numbers ARC/AHRI/117/25).

### Sampling area

**Fig. 1:**
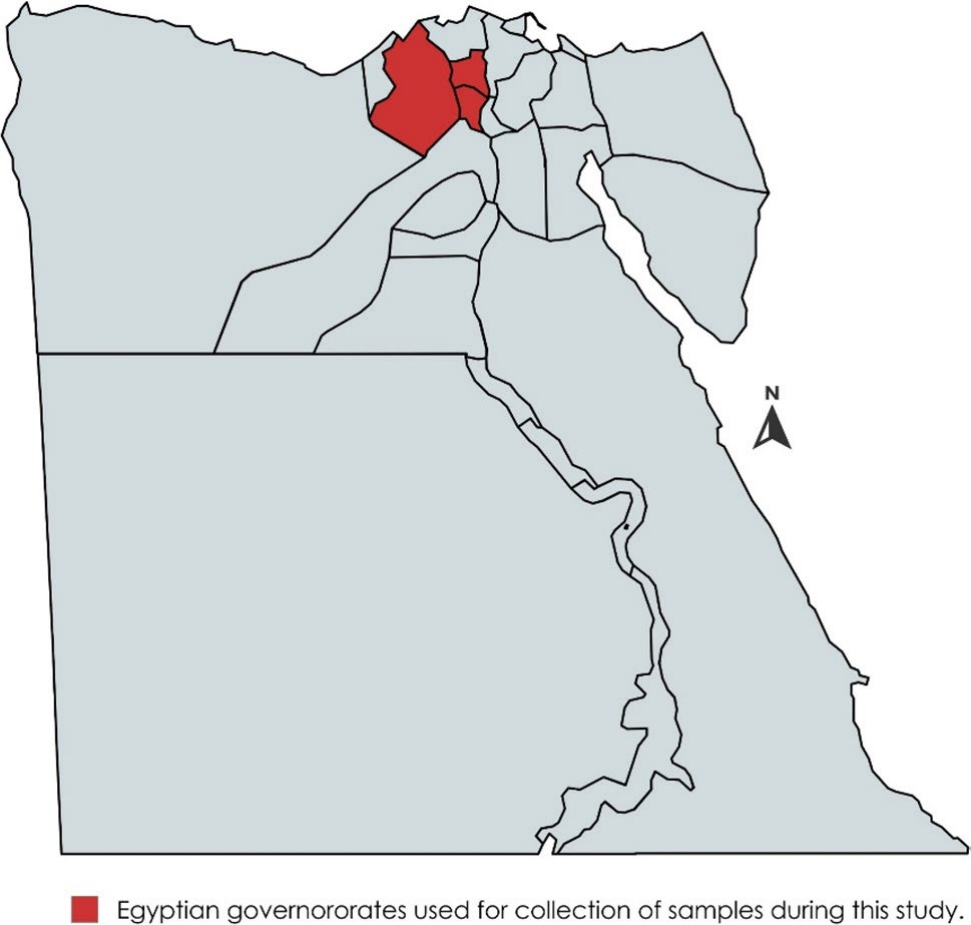
Egypt map shows the location of the governorates (red color) used for sample collection

Cattle farms at 3 Egyptian governorates including Monufia (30.52°N 30.99°E), Gharbia (30.867°N 31.028°E), and Behira (30.61°N 30.43°E) located at the north of Egypt was used in this study (Fig. 1). Physical examination of the animals was done. The history of the tested animals was obtained specially vaccination against LSD vaccine. The animals were of different ages, sex, and breeds.

### Collection of samples

A total number of 168 tissue samples and scabs were obtained by the veterinarian supervising the farms from animals demonstrating LSD signs. Skin nodules and surrounding area were shaved and cleaned well. A local infiltration anesthesia was applied using 1 mL of lidocaine hydrochloride 2% solution, a disposable scalpel was used to have biopsy specimen aseptically. The biopsy specimen and scabs were kept in transport media at ice box with ice bags, and transported to the lab for immediate processing [24, 25].

Parallelly, EDTA vacutainer tubes and plained ones were used for having whole blood samples, and serum samples, respectively through jugular vein puncture. The whole blood samples were divided into 2 parts, the first one was used for measuring the hematological parameters while the 2nd part was preserved at -80 °C until being utilized for nucleic acid extraction. Serum samples were collected either from infected or apparently healthy control ones (the same ages, sex, and breeds. They kept no obvious clinical signs at collection and post collection times) and preserved at -20 °C until being used for measuring biochemical parameters. Separately, blood samples were obtained from 60 healthy animals and used as controls.

### Cells, media, and reagents

Madin Darby Bovine Kidney (MDBK) cells obtained from (VACSERA, Egypt) were used for virus isolation. The cells were grown on Minimum Essential Medium (MEM) with Earle’s Balanced salts and 2.0 mM L-Glutamine (Alliance Bio, Inc.) supplemented with 10% Newborn calf serum (GIBCO, New Zealand) ^®^, antibiotic solution (100 I.U. penicillin, 100 mg streptomycin /ml). Meanwhile pH was kept between 6.8 and 7.2 using Sodium bicarbonate solution [26].

Reference Hyperimmune Serum against *LSDV* (Plum Island, USA), and antibovine IgG conjugated with fluorescein isothiocyanate (Veterinary Laboratory Agency Newha) were used in IFAT.

### Virus isolation

The obtained skin nodules were processed aseptically for virus isolation. After centrifugation, the filterated supernatant was diluted in a ratio of 1:10 with Eagle’s minimum essential medium (E-MEM). T25 flasks cultivated with MDBK cell line were infected with the prepared inoculum and left for 1 h for adsorption time followed by adding the E-MEM and incubation for 5 days. One flask was kept as contol non infected one. The flasks were monitored daily. In case of absence of CPE, the flasks were frozen and thawed for 3 times and used for inoculation of new flasks for 3 successive cycles [27].

### Antigenic characterization by Indirect Fluorescent Antibody Technique (IFAT)

This technique was applied as previously described [25]. The obtained virus isolates were used for infection of tissue culture cells previously grown on cover-slips as well as positive and negative controls. Then, they were fixed with cold acetone and kept at 4 °C. Diluted LSD reference sera in PBS (1/20 or 1/40) was used as a first Ab. and incubated at 37 °C for 1 h. After washing thrice, slides were incubated with the antibovine IgG conjugated with fluorescein isothiocyanate (Veterinary Laboratory Agency Newha) as 2ndry antibodies. Finally, the slides were washed and examined with fluorescent microscope [28].

### Molecular identification

#### Nucleic acid extraction

The preserved whole blood, and tissue samples were used for whole genomic DNA extraction using the GF-1 Nucleic Acid Extraction Kit (Vivantis) according to the instruction manual directions.

The extracted nucleic acid purity and concentration were assessed using a Nanodrop (Thermo Fisher Scientific). The resulting DNA was stored at − 80 °C for molecular detection.

#### Molecular detection by PCR

The conventional PCR was used for molecular detection of *LSDV* using the GPCR, and EEV glycoprotein genes specific primers (Table 1), separately. For each tested samples, the PCR mix consisted of 12.5 µl 2X Emerald Amp Max PCR Master Mix (Takara, Japan), 20 pmol of each primer, DNA template, RNASE free water for a total 25 µl reaction. The reaction was done in BIORAD thermal cycler. The PCR thermal programs for the GPCR, and EEV glycoprotein genes were carried out as previously published [19, 20]. The obtained amplicons were electrophoresed using 1.5% stained agarose gel [26, 29].

**Table 1:** Primers nucleotide sequences, target genes, amplicon sizes used in the current research

Gene name	Primer name	Primer sequence (5′-3′)	Annealing temp.(°C)	Amplicon size (bp)	Reference
GPCR	GPCR-F	TTAAGTAAAGCATAACTCCAACAAAAATG	50 ˚C	1190	(Seerintra et al. 2022) [20]
	GPCR-R	TTTTTTTATTTTTTATCCAATGCTAATACT			
EEV Glycoprotein	EEVGly F	ATGGGAATAGTATCTGTTGTATACG	55 ˚C	958	(Chibssa et al. 2021) [19]
	EEVGly R	CGAACCCCTATTTACTTGAGAA			
DNA-ligase-like (LD133)Wild type	WTR_Frw	GGAATCTGTGCAGAAATAAAGTACGA	60 ˚C	-	(Haegeman et al., 2023) [33]
	WTR_Rev	CCGAAGGGAACGCACTG			
	WTR_Pr	FAM-+CTCATCAAATCCCTCTATTTTATG-TAMRA			
kelch-like (LD144) vaccine type	VR_Frw	GGATTTATTTATATTGTGGGTGGAATT			
	VR_Rev	TTTTTGTATGTCGTAATGGGTTC			
	VR_Pr	HEX-CTCTCGGAATAGGCTATGAAGG-TAMRA			

### Sequencing and phylogeny

The obtained amplicons were purified from the gel using the QIAquick Gel Extraction Kit – Gel Purification Kit (Qiagen, Germany)^®^ and their concentration were measured. Representative samples were sent for sequencing. The assembly and editing of the obtained sequences were conducted using the free Bioedit 7.2 tool ( https://bioedit.software.informer.com/7.2/). The edited sequences of the GPCR gene were uploaded to the National Center for Biotechnology Information (NCBI) database under the accession numbers (PX437384, PX437385). While that for the EEV glycoprotein genes were uploaded (PX437386, PX437387). The Basic Local Alignment Search Tool (BLAST) of the (NCBI) https://blast.ncbi.nlm.nih.gov/Blast.cgi was used for the comparison with the similar sequences.

The phylogenetic analysis was inferred using the minimum evolution method [30]. The evolutionary distances were computed using the maximum composite likelihood method [31] using the MEGA 7 software [32].

### Duplex DIVA qPCR

This technique was applied to determine whether the isolated *LSDV* belonged to vaccinal strain or related to classical field isolated ones (DIVA real-time PCR). The used primers, and probes were previously designed, and validated based on the conserved regions of a DNA-ligase-like (LD133) gene and a kelch-like protein (LD144) gene [33].The qPCR was done in a total volume of 25 µL, consisting of 12.5 µL PerfectStartTM II Probe qPCR SuperMix (Trans Gen Biotech, China), 50–100 ng of the extracted DNA template, 10 pmol of the four primers, 10 pmol of both probes, and the reaction was completed with nuclease free water. Using the CFX Opus 96 Real-Time PCR System, the following thermal program was applied 1 cycle of 95 °C for 10 min and 50 cycles of 95 °C for 10 s and 60 °C for 30 s [33]. Fluorescence acquisition was performed at the end of each annealing/extension step.

### Hematological, and biochemical examination

The different hematological parameters were measured using the Hospitex Diagnostics (Hema Screen 10, Italy).

Serum samples were colorimetrically analyzed using test kits (Biomereux, France) for calcium according to manufacture instruction, total protein [34], albumin [35], globulin which was calculated as the difference between total protein and albumin, blood urea nitrogen [36], creatinine [37], and serum transaminase “AST and ALT” [38]. The creatine phosphokinase (CK-MM) was measured in full automated biochemistry analyzer (Chemray 240. USSR). Some trace element values including zinc, copper, Sodium (Na), calcium (ca.), inorganic phosphorus (iP), magnesium (Mg) and iron concentrations were determined in serum using atomic absorption spectrophotometry [39]. Some oxidative stress parameters such as catalase (CAT) which was determined by colorimetric method using commercial kits provided by (Bio-Diagnostic, Giza, Egypt) according to the instruction of the enclosed pamphlet. Reduced Glutathione (GSH), hydrogen peroxides (H2O2), nitrogen oxide (NO), and malondialdehyde (MDA) were estimated [40].

### Statistical analysis

Data were compiled and managed using Microsoft Excel. Statistical analyses were done using GraphPad Prism software (version 8.0.2; GraphPad Software, San Diego, CA, USA). The normality of data distribution was assessed using the Shapiro-Wilk test. Detection rate of the *LSDV* in the collected tissue, and blood samples using different primer sets were statistically analyzed using Chi-square and 95% confidence interval. Also, risk factors associated with *LSDV* occurrence in the tested animals were statistically analyzed via Chi-square (*χ²*) for all factors while, Fisher’s exact test was used for factors including sex, production type, management, and vaccination against LSD, with odds ratios (OR) and 95% confidence interval (CI) computed to quantify associations and the significance difference was determined at *p* < 0.05. The normally distributed data of serum biochemical and hematological analyses were expressed as mean ± standard error and the statistically analyzed using t-test. Statistical significance was determined at *p* < 0.05; all tests were two-sided.

## Results

### Clinical signs

**Fig. 2:**
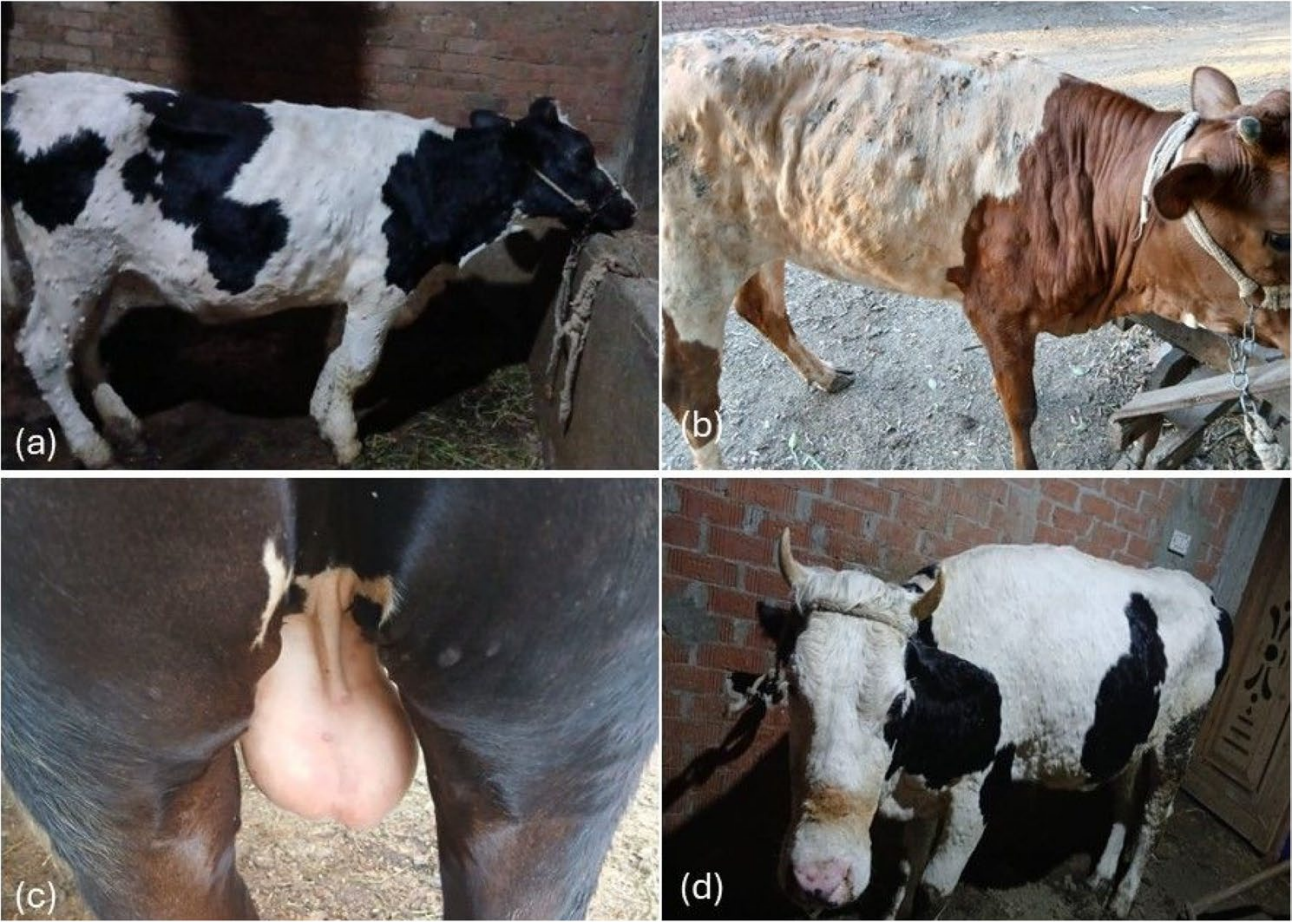
Multiple skin nodules of variant sizes distributed all over the whole body either in the hairy or non-haired areas. The animals were of different ages including young calves and adult ones. **a**, **b** represent young infected calves. **c** presence of skin nodules at the Perineal region, **d** represents adult cow suffered with generalized skin nodules

Out of the 168 animals examined, the most prominent clinical signs related to LSD were hyperthermia as temperature varied from 39.6 °C to 40.5 °C, and the development of multiple skin nodules of variant sizes usually distributed all over the whole body either in the hairy or non-haired areas. Some nodules progressed into necrotic changes complicated with secondary bacterial infection. Lacrimation of the eye, and enlargement of the regional lymph nodes were also determined (Fig. 2).

### Virus isolation

**Fig. 3:**
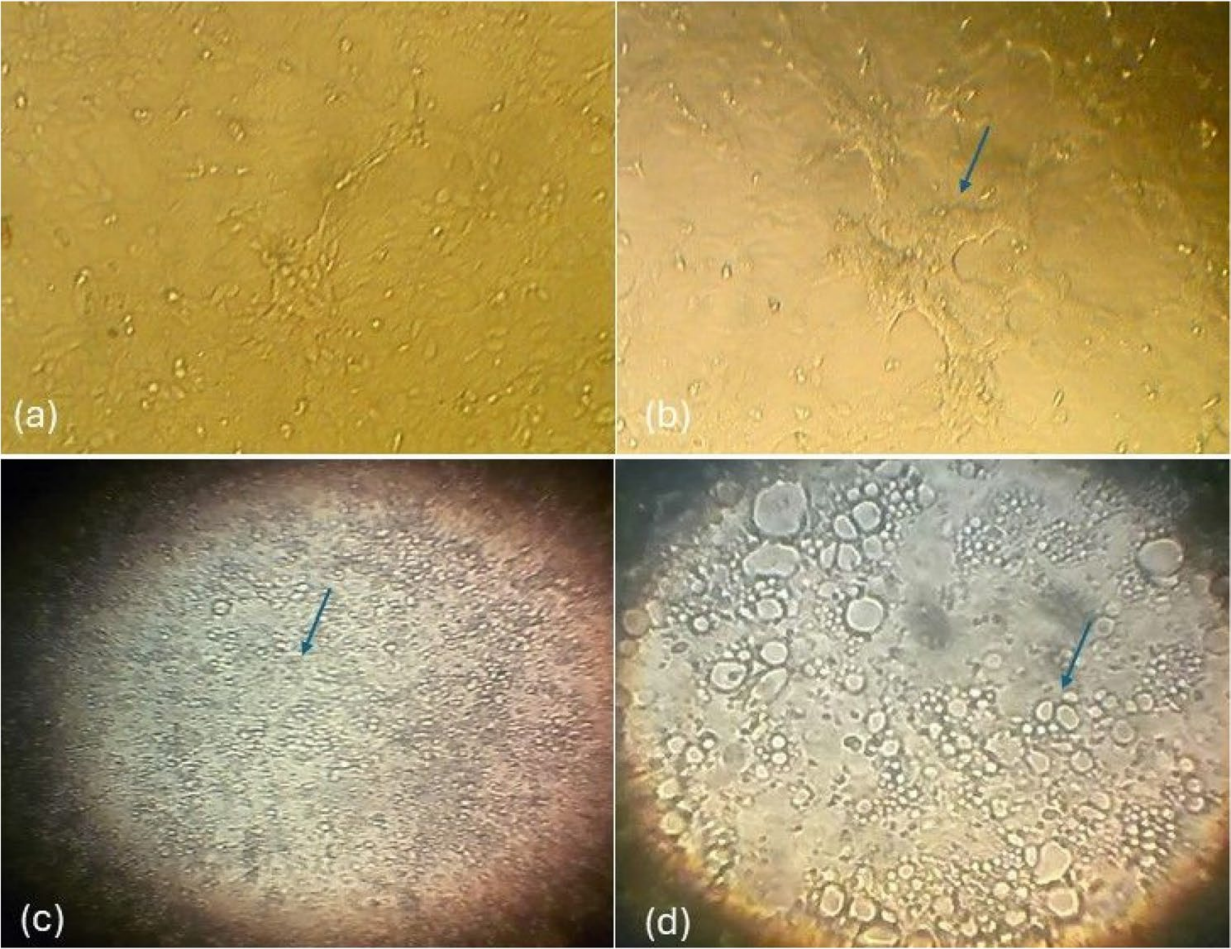
Isolation of *LSDV* on MDBK cells. The collected skin nodules were processed and subjected to virus isolation on MDBK. **a** normal MDBK cells kept as negative control. **b** MDBK cells infected with processed skin nodules started to show CPE in the form of cell rounding, clusters and cell aggregation. **c** MDBK cell vacuolation after 72 h of infection. **d** distributing vacuoles across the MDBK sheet in 4th and 5th day post infection

The collected skin nodules were processed and subjected to virus isolation on MDBK for 3–5 blind passages. Mostly, CPE appeared after 3–4 blind passages. After 3 days of inoculation, the CPE developed in the form of cell rounding, clusters and cell aggregation, followed by detachment of the monolayer sheet (Fig. 3). Although it was not applicable to isolate all field samples but a total ratio of 38.69% (65/168) *( 95% CI: 31.3–46.1*) was recorded. Menoufia has a low positivity rate of 33.80% followed by Gharbia (39.47%), and Behira (44.06%) (Table 2).

**Table 2:** Prevalence rate of the LSDV in the collected tissue, and blood samples using different primer sets

Total collected samples		Virus isolation			Molecular detection by GPCR gene			Molecular detection by EEV Glycoprotein			Statistical indices	
		positive	%	(95 % CI)	Positive	%	(95 % CI)	Positive	%	(95 % CI)	*P* value	*X* ^2^
Tissues samples												
Menoufia	71	24	33.80	(31.3-46.1)	63	88.73	(82.6-92.0)	60	84.50	(74.3-86.3)	<0.0001	62.04
Gharbia	38	15	39.47		33	86.84		28	73.68		<0.0001	20.45
Behira	59	26	44.06		52	88.13		48	81.35		<0.0001	32.39
Total	168	65	38.69		148	88.09		136	80.95		<0.0001	112.5
Blood samples												
Menoufia	71	Not applicable			44	61.97	(60.2 –74.3)	38	53.52	(49.6–64.6)	0.395	1.03
Gharbia	38				26	68.42		19	50		0.160	2.67
Behira	59				43	72.88		39	66.10		0.549	0.63
Total	168				113	67.26		96	57.14		0.071	3.65

The prevalence is significantly different using Chi square test when *p*<0.05, highly significant *P*<0.01, highly significant *P*<0.0001

### Characterization by IFAT

**Fig. 4:**
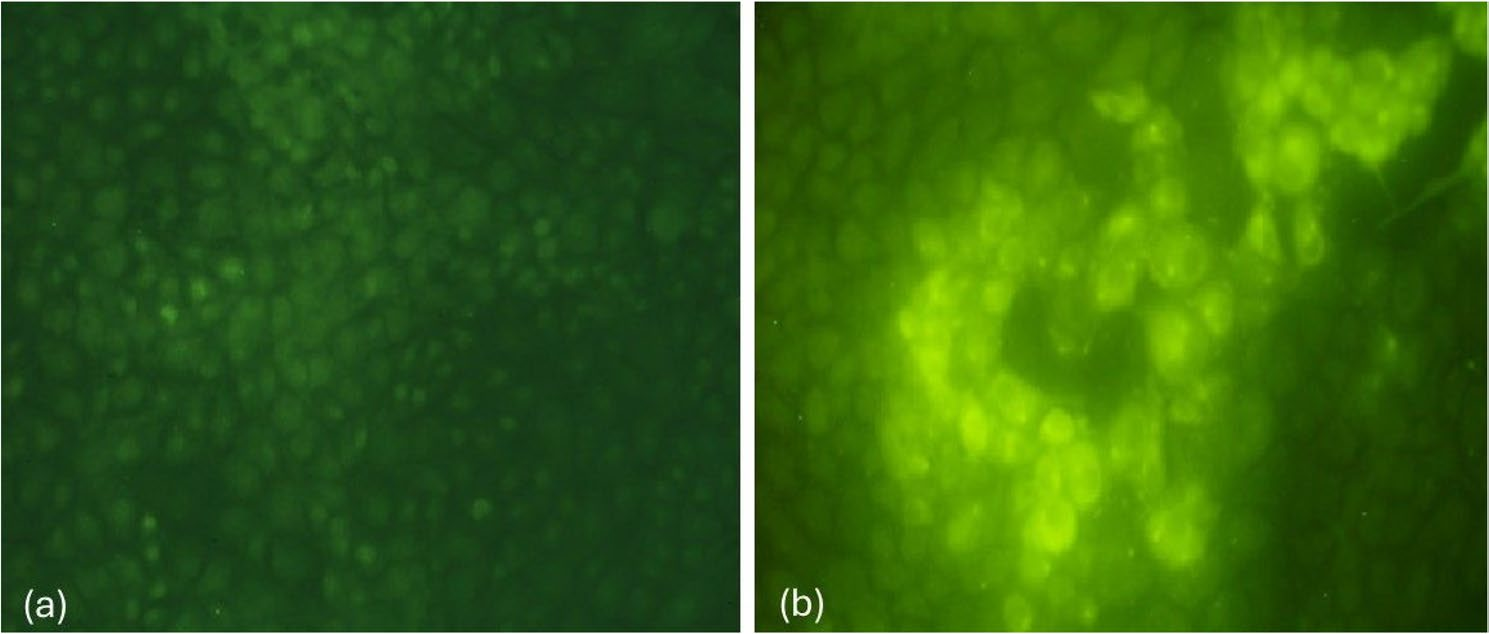
Serological confirmation of the obtained *LSDV* isolates using the IFAT. MDBK cells were infected with the obtained *LSDV* isolates. After appearance of CPE, fixation was done. Diluted *LSDV* reference sera, and antibovine IgG conjugated with fluorescein isothiocyanate were used as primary and secondary antibodies, respectively. **a** the mock non infected cells showed no reaction. **b** the *LSDV* infected cells showed intracytoplasmic fluorescent reactions

All of the obtained virus isolates were subjected to serological confirmation using the IFAT. The obtained results confirmed isolation of LSD as the virus reacted with *LSDV*-specific reference antisera yielding intracytoplasmic fluorescent reactions observed in the tested cells. Meanwhile, the mock non infected cells showed no reaction (Fig. 4).

### Molecular detection

**Fig. 5:**
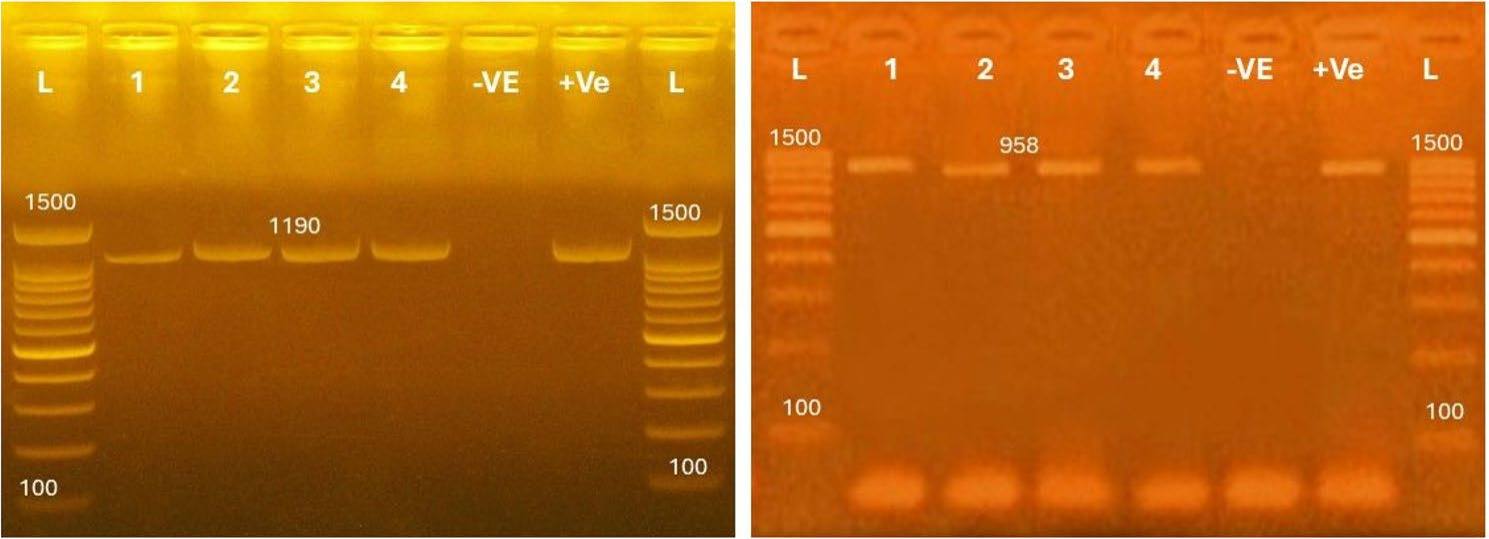
Molecular detection of *LSDV* in the collected blood, and tissue samples. DNA extraction of the obtained blood, and tissue samples was done. Using the GPCR primers set result in amplification of 1100 bp, while using EEV glycoprotein primers revealed amplification of 958 bp

All the collected blood, and tissue samples were subjected to molecular detection by conventional PCR using 2 different sets of primers based on the GPCR genes which result in amplification of 1100 bp, and EEV glycoprotein gene which result in 958 bp (Fig. 5).

The detection rate of *LSDV* in the collected tissue samples varied according to the diagnostic method used. Molecular detection using the GPCR gene primer set showed a higher rate of 88.09% (*148/168; 95% CI: 82.6–92.0*), while detection using the EEV glycoprotein gene revealed a ratio of 80.95% (*136/168; 95% CI: 74.3–86.3)* with presence of significant difference (*χ² = 112.5*,*P* < 0.0001).

Regarding blood samples, the detection rate of *LSDV* by GPCR gene primer set was 67.26% (*113/168; 95% CI: 60.2–74.3*), whereas EEV glycoprotein primer set detected the virus in 57.14% (*96/168; 95% CI: 49.6–64.6*) of the examined samples. No statistically significant difference was observed between the two molecular assays for blood samples (*χ² = 3.65*, *P* = 0.071) (Table 2).

### Duplex DIVA qPCR

**Fig. 6:**
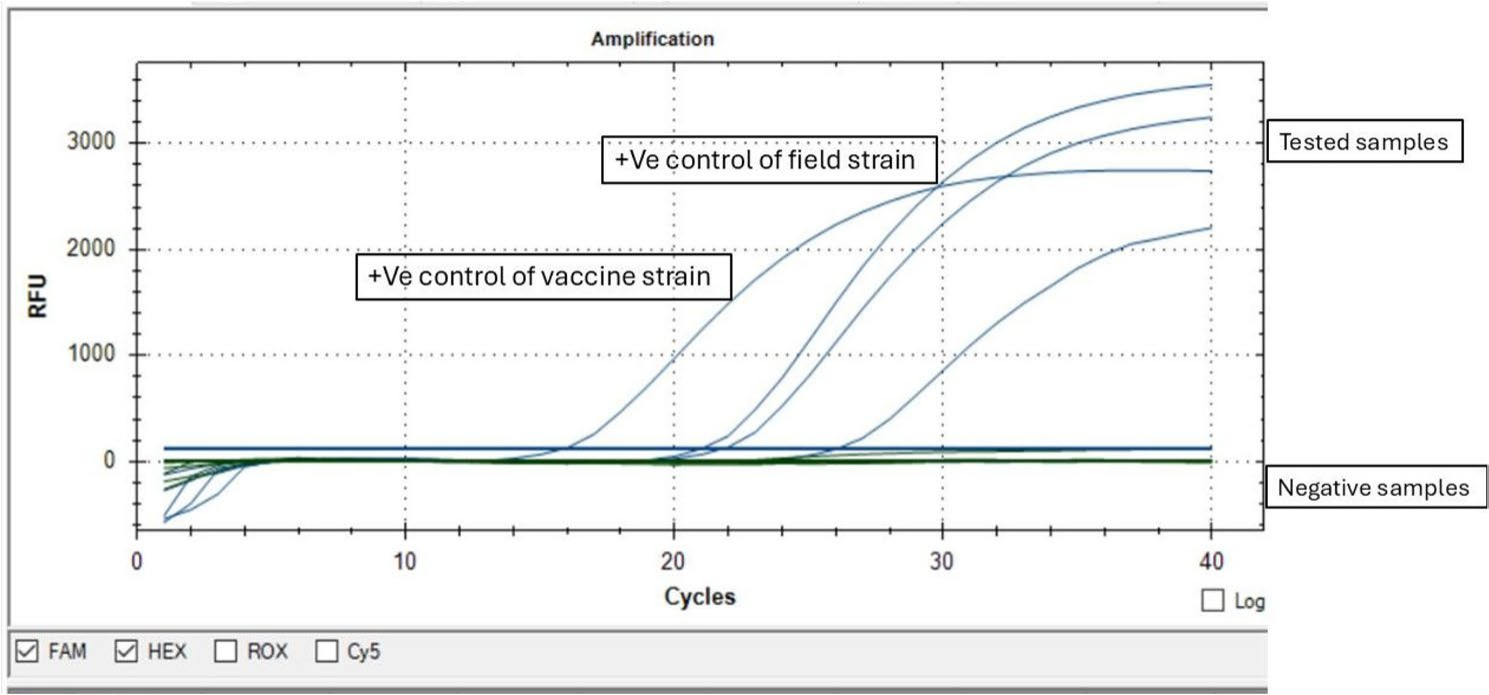
DIVA real-time PCR of the obtained *LSDV*. qPCR was applied for detection of the extracted DNA samples using 2 different sets of primers, and probes representing vaccinal, and field strains. Fluorescence acquisition was performed at the end of each annealing/extension step. All detected samples were related field isolates not vaccinal strains

Due to appearance of clinical symptoms in some vaccinated animals vaccinated with the homologous LSD strain. A duplex DIVA qPCR based on the conserved regions of a DNA-ligase-like (LD133) gene and a kelch-like protein (LD144) gene was applied to determine whether the causative strains were vaccinal or field strains. The results showed that none of the detected strains were due to vaccine, and all were related to field isolates (Fig. 6).

### Phylogeny

The obtained sequences were deposited at the NCBI database under the accession numbers (PX437384, PX437385) for the GPCR gene, while that for the EEV glycoprotein genes were (PX437386, PX437387).

The cladogram analysis revealed that the Capri pox viruses were clearly divided into three distinct lineages, *SPPV*,* GTPV* and *LSDV* where all of the sequences reported in the current research were clustered together in one lineage with other *LSDV* sequences from cattle obtained from GenBank.

**Fig. 7:**
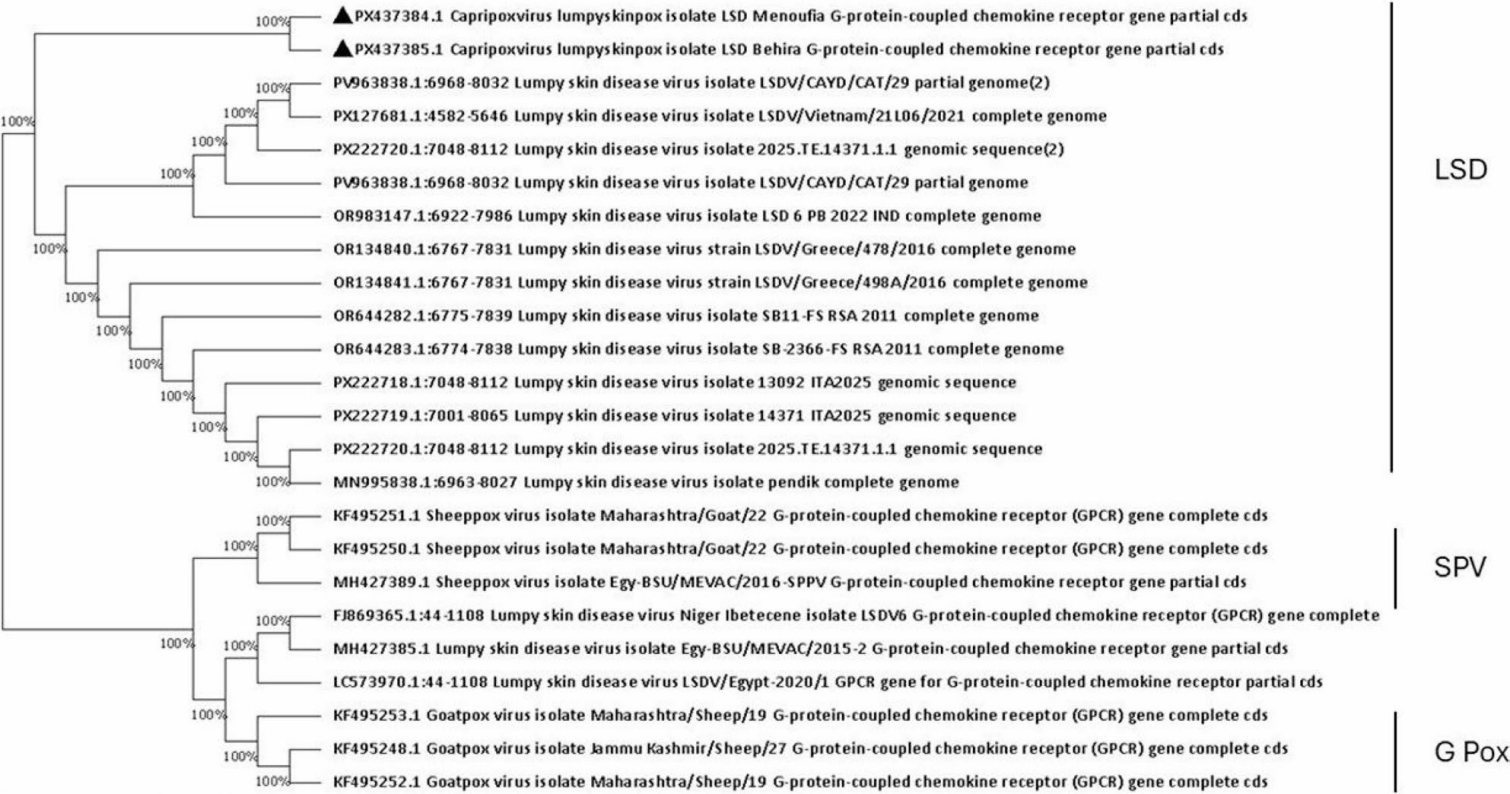
Phylogenetic analysis of the obtained *LSDV* based on the GPCR gene sequence. The obtained GPCR gene amplicons were sequenced and uploaded to the NCBI database. The phylogenetic analysis was inferred using the minimum evolution method. The evolutionary distances were computed using the maximum composite likelihood method. Evolutionary analyses were conducted using the MEGA 7 software. The black triangles represent the obtained sequences of this study (PX437384, PX437385)

The phylogeny based on the GPCR showed that, the obtained sequences were related to each other with no significant difference, also related to the strains recently detected in Nigeria ( PV963838.1), and Vietnam (PX127681) during 2025 (Fig. 7).

**Fig. 8:**
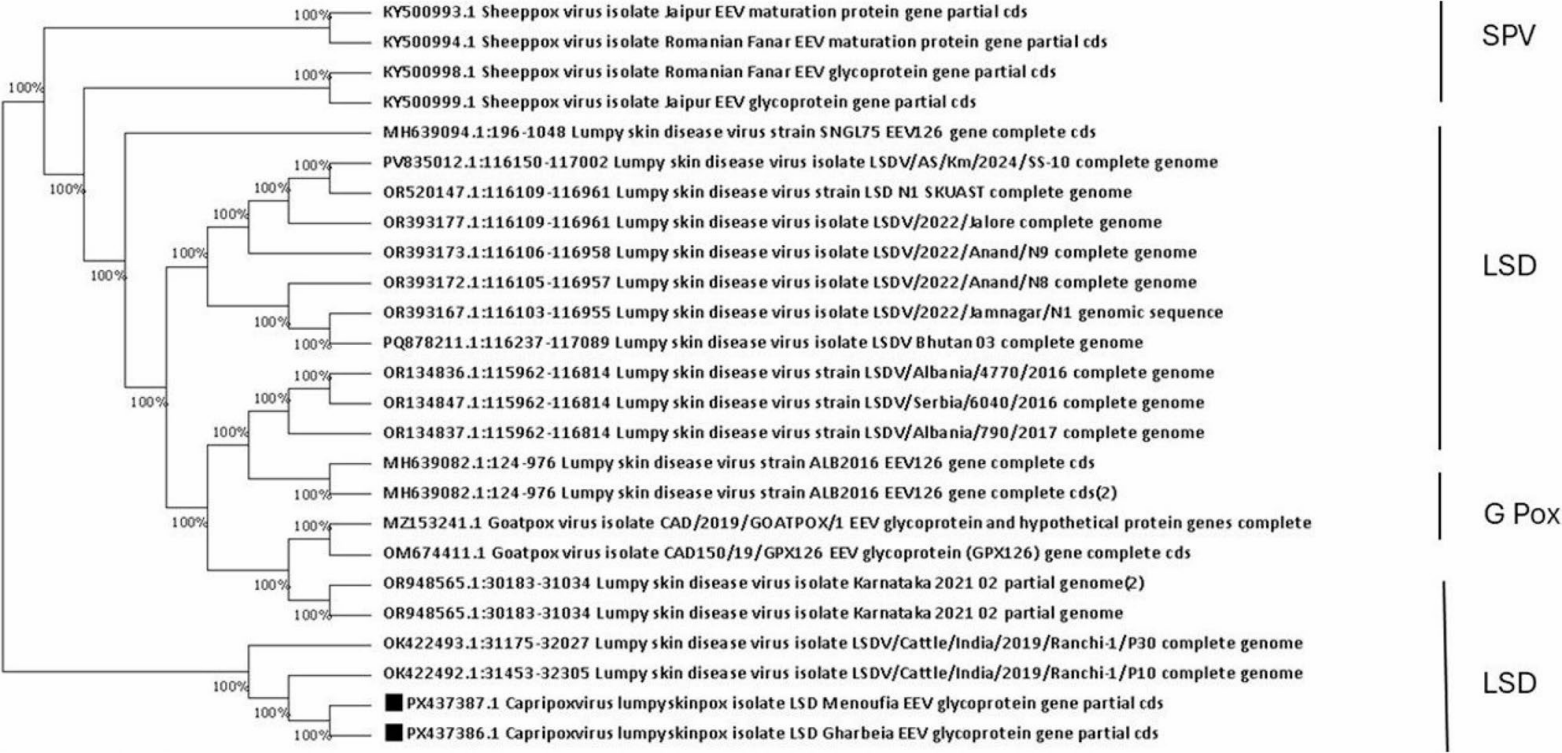
Phylogenetic analysis of the obtained *LSDV* based on the EEV glycoprotein gene sequence. The obtained EEV glycoprotein gene amplicons were sequenced and uploaded to the NCBI database. The phylogenetic analysis was inferred using the minimum evolution method. The evolutionary distances were computed using the maximum composite likelihood method. Evolutionary analyses were conducted using the MEGA 7 software. The black squares represent the obtained sequences of this study (PX437386, PX437387)

Also, the determined isolates based on the EEV glycoprotein gene, the PX437386, PX437387 were related to each other and located within the same clade of LSD with OK422492, and OK422493 detected in India during 2019 (Fig. 8).

### Risk factors assessment

The association between several risk factors and the occurrence of *LSDV* infection among the examined animals is presented in Table 3. Regarding sex, the detection rate of *LSDV* infection was 76.92% (40/52) in males and 93.10% (108/116) in females with presence of statistically significant association between sex and *LSDV* infection **(***χ² = 8.96*, *P* = 0.004. The calculated odds ratio indicated that males had lower odds of infection compared to females (*OR = 0.24; 95% CI: 0.09–0.63*). Concerning age, the infection rate was highest in animals aged 0–6 months (100%; 67/67), followed by animals aged 6 months–2 years (84.84%; 56/66), while the lowest detection rate was recorded in animals aged ≥ 2 years (71.42%; 25/35). A highly significant difference was observed among age groups (*χ² = 18.99*, *P < 0.0001*). Regarding to breed, the detection rate was 81.25% (52/64) in local breeds, 98.24% (56/57) in Friesian cattle, and 85.10% (40/47) in mixed breeds. Breed was significantly associated with *LSDV* occurrence (χ² = 8.86, *P* = 0.01). Regarding production type, *LSDV* detection was 89.13% (82/92) in beef cattle and 86.84% (66/76) in dairy cattle, with no significant difference between the two groups (*χ² = 0.20*, *P = 0.81; OR = 1.24; 95% CI: 0.47–3.27*). Similarly, management system showed no statistically significant association with *LSDV* infection. The detection was 84.12% (53/63) in intensive management systems compared to 90.04% (95/105) in small-holder farms (*χ² = 1.51*, *P = 0.22; OR = 0.55; 95% CI: 0.21–1.48*). A highly significant association was observed between vaccination status and *LSDV* infection as the non-vaccinated animals showed a markedly higher rate (96.26%; 129/134) compared with vaccinated ones (55.88%; 19/34) (*χ² = 42.18*, *P < 0.0001*). The odds of infection were significantly higher in non-vaccinated animals (*OR = 20.37; 95% CI: 6.56–54.00*). Regarding location, the detection rate of *LSDV* was 88.73% (63/71) in Menoufia, 86.84% (33/38) in Gharbia, and 88.13% (52/59) in Behira, with no significant difference among the governorates (*χ² = 0.08*, *P = 0.95*). Finally, the determination across the years of the study was 86.36% (38/44) in 2023, 87.50% (42/48) in 2024, and 89.47% (68/76) in 2025, with no statistically significant difference among the years (χ² = 0.27, *P = 0.86*).

**Table 3:** Risk factors associated with LSDV occurrence in the tested animals of Egyptian governorates

Tested risk factors		Examined	Positive	%	P value	X^2^	OR	(95 % CI)
Sex	Male	52	40	76.92	0.004	8.96	0.24	(0.09 - 0.63)
	Female	116	108	93.10				
Age	0- 6 M	67	67	100	<0.0001	18.99		
	6M-2 Y	66	56	84.84				
	≥ 2 Y	35	25	71.42				
Breed	Local bread	64	52	81.25	0.01	8.86		
	Friesian	57	56	98.24				
	Mixed Sp.	47	40	85.10				
Production type	Beef	92	82	89.13	0.81	0.20	1.24	(0.47 - 3.27)
	Dairy	76	66	86.84				
Management	Intensive	63	53	84.12	0.22	1.51	0.55	(0.21 – 1.48)
	Small holder	105	95	90.04				
Vaccination against LSD	Non-Vaccinated	134	129	96.26	<0.0001	42.18	20.37	(6.56 - 54.00)
	Vaccinated	34	19	55.88				
Location	Menoufia	71	63	88.73	0.95	0.08		
	Gharbia	38	33	86.84				
	Behira	59	52	88.13				
Years	2023	44	38	86.36	0.86	0.27		
	2024	48	42	87.50				
	2025	76	68	89.47				
Total		168	148	88.09				

Means are significantly different at *p*<0.05, the *OR* odds ratio for Fisher's exact test and *X*^2^ for Chi-square test and *CI* Confidence interval at 95%

### Biochemical and hematological investigations

The biochemical investigation of *LSDV* infected animals cleared a marked decline in the total protein (TP) as it was 6.49 ± 0.48 compared to 8.23 ± 0.42 in healthy one (*P < 0.01*), Albumin (3.55 ± 0.20 and 2.69 ± 0.15), and Globulin (4.68 ± 0.26 and 3.80 ± 0.20) in healthy and infected animals, respectively with significant difference (*P < 0.05*). Liver enzyme activities were significantly (*P < 0.05*) elevated in LSD-infected animals as ALT increased from 16.25 ± 1.12 IU/L in healthy animals to 25.44 ± 1.23 IU/L in infected cases, while AST increased from 39.05 ± 1.20 IU/L to 47.39 ± 1.11 IU/L (P < *0.05*). Furthermore, renal function markers also showed significant increases, where urea levels rose from 40.5 ± 0.62 mg/dl in healthy cattle to 52.20 ± 2.15 mg/dl in infected animals (*P < 0.05*). Creatinine levels similarly increased from 1.05 ± 0.06 mg/dl to 1.97 ± 0.23 mg/dl (*P < 0.05*). Creatine kinase (CK-MM) activity showed a significant elevation in infected animals (265.6 ± 2.88 U/L) compared with healthy cattle (249.4 ± 4.02 U/L) (*P < 0.05*). Electrolyte analysis revealed a significant decrease in sodium levels in infected animals (95.62 ± 0.56 mmol/L) compared with the healthy group (125.23 ± 0.99 mmol/L) (*P* < 0.05). Calcium, inorganic phosphorus, and magnesium concentrations were also significantly reduced in infected cattle (*P < 0.05*). Regarding oxidative stress markers, catalase activity (CAT) showed a highly significant decrease in infected animals (150.21 ± 0.12 U/L) compared with healthy animals (351.23 ± 0.15 U/L) (*P < 0.01*). Reduced glutathione (GSH) levels were also significantly decreased (*P < 0.05*). Conversely, oxidative stress indicators including malondialdehyde (MDA), hydrogen peroxide (H₂O₂), and nitric oxide (NO) showed highly significant increases in infected animals (*P < 0.01*) (Table 4).

**Table 4:** Serum biochemical parameters in apparently healthy and diseased animals suffering from LSD (mean ±SE)

Parameters	Apparently health (*n*=60)	LSD infected animals (*n*=168)
T. protein gm/dl	8.23±0.42	6.49±0.48**
Albumin gm/dl	3.55±0.20	2.69±0.15*
Globulin gm/dl	4.68±0.26	3.80±0.20*
ALT IU/L	16.25±1.12	25.44±1.23*
AST IU/L	39.05±1.20	47.39±1.11*
Urea mg/dl	40.5±0.62	52.20± 2.15*
Creatinine mg/dl	1.05±0.06	1.97±0.23*
CK-MM (U/L)	249.4±4.02	265.6± 2.88*
Sodium (mmol/L)	125.23±0.99	95.62±0.56*
Calcium (mg/dl)	11.15±0.42	9.11±0.35*
iP (mg/dl)	5.11±0.11	3.88±0.03*
Mg(mg/dl)	3.52±0.02	2.12±0.05*
CAT (U/L)	351.23±0.15	150.21±0.12**
GSH (mmol/ml)	25.25±0.21	12.68±0.30*
MDA (mmol/ml)	3.06±0.06	11.84±0.23**
H2O2 (ng/ml)	290.25±0.23	601.5 ± 0.36**
NO (ng/ul)	31.02±1.05	59.16±0.54**

The mean values is considered Significant using t-test *P*<0.05 **highly significant *P*<0.01 and ***highly significant *P*<0.001

*CK-MM* Creatine phosphokinase, *GSH* Reduced Glutathione, *H2O2* hydrogen peroxides, *NO* nitrogen oxide, and *MDA* malondialdehyde

The hematological findings were summarized in (Table 5) as *LSDV* infection resulted in significant hematological alterations. A significant decrease in erythrocytic parameters was observed in infected animals. RBC count decreased from 7.98 ± 0.21 × 10⁶/µl in healthy animals to 6.02 ± 0.25 × 10⁶/µl in infected animals (*P < 0.05*). Hemoglobin concentration was highly significantly reduced from 12.94 ± 0.15 g% to 9.35 ± 0.20 g% (*P < 0.01*), while packed cell volume (PCV) decreased from 34.11 ± 0.55% to 31.03 ± 0.42% (*P < 0.05*). The Mean corpuscular volume (MCV) showed a significant increase in infected animals (51.54 ± 0.33 fl.) compared with healthy animals (42.74 ± 0.24 fl.) (*P < 0.05*), while MCH did not show a significant difference between the two groups. Mean corpuscular hemoglobin concentration (MCHC) showed a highly significant decrease in infected cattle (30.13 ± 0.52%) compared with the healthy group (37.39 ± 0.36%) (*P < 0.01*). Leukocytic parameters revealed a highly significant decrease in total leukocyte count in infected animals (7.64 ± 0.39 × 10³/µl) compared with healthy cattle (11.6 ± 0.40 × 10³/µl) (*P < 0.01*). Lymphocyte count also showed a highly significant reduction in infected animals (3.18 ± 0.23 × 10³/µl) compared with healthy animals (7.02 ± 0.22 × 10³/µl) (*P < 0.01*). However, neutrophils, monocytes, eosinophils, and basophils did not show significant differences between the two groups.

**Table 5:** Erythrogram parameters in healthy and diseased animals suffering from LSD (mean and ±SE)

Parameters	Apparently health (*n*=60)	LSD infected animals (*n*=168)
RBCs count (*10^6^/ul)	7.98±0.21	6.02±0.25^*^
Hb (g%)	12.94±0.15	9.35±0.20^**^
PCV(%)	34.11±0.55	31.03±0.42*
MCV(fl)	42.74±0.24	51.54±0.33^*^
MCH(pg)	16.21±0.16	15.81±0.18
MCHC(%)	37.39±0.36	30.13±0.52^**^
WBCs(10^3^/ul)	11.6±0.40	7.64±0.39^**^
Neutrophil(10^3^/ul)	3.08±0.11	3.02±0.15
Lymphocyte(10^3^/ul)	7.02±0.22	3.18±0.23^**^
Monocyte(10^3^/ul)	1.33±0.09	1.20±0.08
Esinophile(10^3^/ul)	0.13±0.03	0.18±0.06
Basophil(10^3^/ul)	0.04±0.02	0.06±0.02

The mean values is considered Significant using t-test *P*<0.05, **highly significant *P*<0.01 and ***highly significant *P*<0.001

## Discussion

Lumpy skin disease caused by the *LSDV* is a vector- borne diseases that has significant drastic effect on infected animal health. Investigating the clinical cases revealed presence of multiple signs but mainly fever, and skin lesions were prominent. The same results were previously reported for infection with this disease due to many field isolates. The lesions could appear after an incubation period ranged from 7 to 30 days [14]. Although *LSDV* has a wide tropism to the dermal tissue the severity of the nodular lesions varied according to many factors including mainly the infectious virus strain [15]. These lesions could be contaminated by secondary bacterial infection which result in bad prognosis or even death of many cases specially in the non-immunized young ages [25].

Virus isolation is the golden standard technique for diagnosis [8, 41]. So, the trial for *LSDV* isolation was successfully done from the collected tissue samples. The infected cells showed cell rounding, aggregations, and vacuolation, the same CPE was previously demonstrated during *LSDV* isolation [27]. Number of the required blind passages, and severity of CPE depend mainly on virus strain, viral load, and type of the collected samples. Although the virus could be secreted and detected in many body secretions, but it is advisable to isolate the virus from the skin nodules before secondary bacterial contamination due to presence of high viral load [15]. Although only 38.69% of the collected samples were isolated but this could be attributed to the method of sampling, handling, and type of used cells. The virus was previously isolated and propagated on different cell lines [42]. All of the obtained virus isolates were confirmed by IFAT. This tool was widely used for antigen detection due its easy application, and reasonable high accuracy [27]. Although for antibody detection, VNT could be superior [25].

Developing of molecular tools was a promising approach for identification, and characterization of many pathogens [43, 44]. Although many primer sets were used for molecular detection of *LSDV* but those developed based on the GPCR, and EEV glycoproteins genes were commonly used in previous studies. The obtained results cleared the comparative superiority of the GPCR gene specially in the collected tissue samples rather than blood [20].

This result could be attributed to the high tropism of the *LSDV* to dermal tissues. Consequently, a high particle count could be found and survive for long times. On the other hand, for detection of virus in blood, optimum sample collection at the viremic status is mandatory [15]. The obtained molecular detection rate varied from 80.95% − 88.09%, which was more accurate than the traditional virus isolation method but inability to detect some samples might been caused by technical errors, insufficient sample DNA processing, or poor preservation conditions [45].

DIVA strategy is commonly used to differentiate between infected, and vaccinated cases based on serological and molecular techniques [46]. In the current study, all detected virus samples belonged to field strains rather than vaccinal ones. The used primer set was confirmed to differentiate between the Neethling-based vaccine strains, field, and recombinant wild-type based on DNA-ligase-like (LD133) gene and a kelch-like protein (LD144) gene. Such approach is important to understand the causes of outbreaks. It helps in root cause analysis which is essential to control the outbreaks [33]. But determination of infection in previously vaccinated animals raises the concern about the used vaccine efficiency, immunization protocol, handling of vaccine, and the immunization method, all of these factors should be well investigated to determine the root cause analysis [11].

Although many genes including P32 were previously used for molecular detection and sequencing [18], but the GPCR gene has a variable nucleotide sequence. Therefore, it is easily used for differentiation between the *Capri pox* viruses or those field and vaccinal strains. The phylogenetic analysis confirmed genetic relatedness between the sequenced isolates, and those previously determined in India, Nigeria, and Vietnam. Appearance of *LSDV* outbreaks usually occurs due to infected animal trade between the countries [22]. But actually, Egypt did not import live animals from these countries before or during study which raise the concern of transportation methods including vectors which are mainly responsible for a short-distance spread [47], or other methods that need to be deeply investigated.

Age, sex, and breed were found to be significant risk factors correlated with the infection rates. The same notice was previously concluded from epidemiological investigation of different outbreaks in Turkey [48]. The young animals were found to be highly infected compared to the older ages. This might be due to the farmer concept not to vaccinate animals in ages [49]. Also, older animals could have memory immune cells that help in rapid initiation of response upon infection. This data also highlights the importance of vaccination time of the newly born calves specially those having passive immunity from their immunized dams which could last for 12–14 weeks [50]. Conversely, these results were in contrast to that recently recorded in Bangladesh [9]. Females were found to be subjected to high infection rate compared to males. This might be due to the multiple physiological stress factors including lactation or pregnancy period that lowers immunity. Also, the females were subjected to high incidence of infection as they were kept for longer times compared to males which fattened and slaughtered almost at age of 2 years [48].

The vaccinated animals were significantly less infected than non-vaccinated ones. The same observation was recently reported during cattle outbreak in Bangladesh [9]. Although Egypt applies massive vaccination strategy based on the homologues Neethling strain which was proven to be the best choice, but other countries depend on application of heterologous sheep pox or goat pox vaccines due to the concept of safety profile, and avoiding recombination process [11]. Detection of infection in some vaccinated animals could be due to failure of the vaccination process or the individual variance in immune response. Furthermore, it raises the concern of further mandatory interventions to be applied as a learned lesson from failure of vaccination strategy in Turkey during 2012 [51].

Regarding the erythrogram, a significant decline was determined in the total erythrocytic count and hemoglobin concentration of the infected cases. *LSDV* infection not only affected clinical situation of infected cattle but also affected significantly internal hemogram including reduced RBCS count, Hb content, HCT, and MCHC values with a significant increased MCV values in all LSD infected animals compared to healthy control ones [52, 53]. These alterations indicated macrocytic hypochromic anemia due to destruction of RBCs as it increases production of negative oxygen species which have negative effect on membrane of RBCs cause osmotic fragility and destruction of cell membrane. This result revealed as a macrocytic hypochromic anemia which could occur with viral infection [61].

Leukogram analysis revealed marked leucopenia and lymphopenia during the early infection [17, 52] which is mainly returned to viral infection especially during viral pathogenesis including systemic spread and environmental viral shedding. These results may be attributed to release of large quantities of endogenous corticosteroid [54]. Significant elevated serum activities of ALT, ALP and AST were found during infection compared to healthy control animals which may be due to primary hepatic injury by *LSDV* or secondary to anoxic necrosis of periacinar hepatocytes (Cockcroft) [55]. AST activity also may be elevated due to affection of cardiac muscles [56]. Additionally, there was a highly significant increase in blood urea nitrogen in diseased animals in comparison with the apparently healthy groups as reported [57].

Cattle infected with LSD showed a significant elevation in urea and creatinine compared to healthy animals. This elevation could be attributed to increased catabolism of protein and virus effect on kidneys [57].

The significant increase of CK in LSD infected cattle could be due to muscle damage involvement [58]. Calcium, magnesium, and phosphate are important for many biologic and cellular functions. The cause of hypocalcemia may be associated with hypoalbuminemia as about one-half of all calcium is attached to serum albumin [59]. The kidneys play a major role in the homeostasis of these ions [60]. Also, reduced levels of ca., iP, Mg and Na may indicate kidney dysfunction. At the same time, hyponatremia could refer to reduction in food intake or decreased absorption [61].

Reactive oxygen species (ROS) and nitric oxide (NO) are produced during the infection as a response of animal innate immunity to infectious agent. It has a destructive effect to cell protein, cell membrane lipid and nucleic acid resulting in lipid peroxidation and cell destruction [62]. Malondialdehyde (MDA) is a lipid peroxide biproduct which is produced during oxidative stress in cell [63]. In this study, LSD infected animals showed significant increase in MDA and NO with significant decrease in CAT and GSH which are indicative to oxidative stress [61]. The results of the present study were in accordance with [64] which reported that cattle infected with LSD showed higher level of MDA and NO along with lower levels of GSH and CAT. Oxidative stress occurs when the amounts of oxygen radical production, such as (H2O2) and (NO), surpass the antioxidant levels, such as catalase and reduced glutathione. This was consistent with the findings of [65], who found that when ROS levels increased, antioxidants decreased.

## Conclusion

Severe outbreaks of *LSDV* infections affected Egyptian cattle in different governorates despite application of vaccine programs with the homologous strains. The causative agent virus was successfully isolated on MDBK cells. The obtained isolates were successfully characterised by IFAT, and PCR. Followed by phylogenetic analysis. Using the GPCR primers, and tissue samples was more accurate in disease diagnosis compared to EEV glycoprotein gene primer set, and blood samples, respectively. All of the detected virus strains were related to field isolates not vaccinal ones. The constructed phylogenetic tree showed the clear distinguishing of *capripox* lineages, and relatedness of the obtained isolates to each other. Sex, age, breed, and vaccine application were the most correlated risk factors. The *LSDV* infection led to alteration of biochemical parameters with the resulted anemia, hypoproteinemia, significant increase in blood urea nitrogen, creatinine, AST, and ALT.

## Supplementary Information

Supplementary Material 1.

## Data Availability

All the data related to this manuscript is available upon request. All sequence data are available: https://www.ncbi.nlm.nih.gov/nuccore/PX437384https://www.ncbi.nlm.nih.gov/nuccore/PX437385https://www.ncbi.nlm.nih.gov/nuccore/PX437386https://www.ncbi.nlm.nih.gov/nuccore/PX437387.
