# Supplementary figures and images for: Lumpy Skin Disease Virus (LSDV) recently emerged in Egypt: molecular detection, and assessment of the related hematobiochemical, and risk factors
Source: BMC Vet Res. 2026 Apr 13;22:230. doi: 10.1186/s12917-026-05442-7 (PMC13085450; doi:10.1186/s12917-026-05442-7)

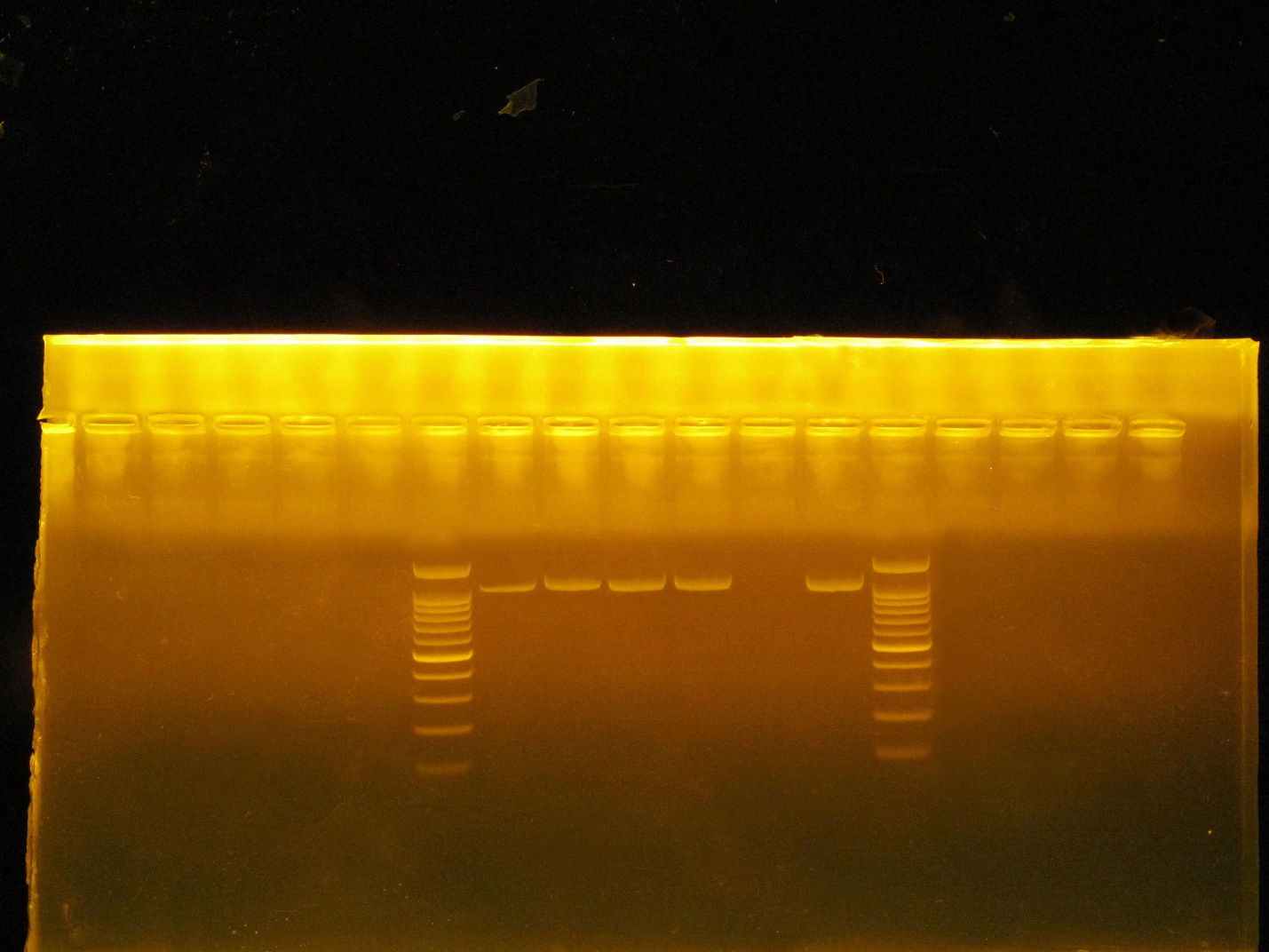


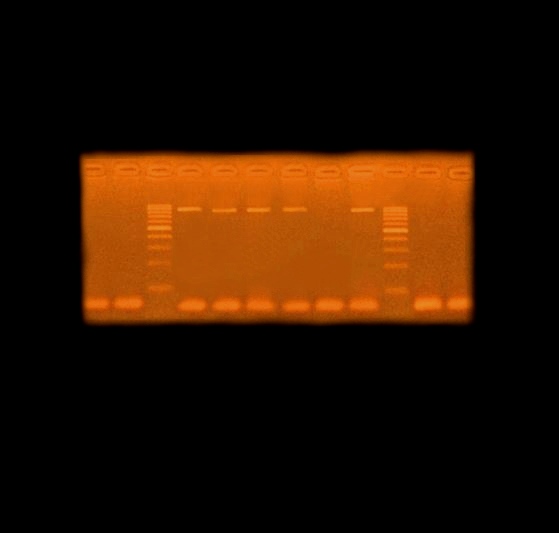

Supplement: Supplementary file 1 — Supplementary Material 1. [file 12917_2026_5442_MOESM1_ESM.docx]
